# A New Enzyme Immunoassay for the Quantitative Determination of Classical Autotaxins (ATXα, ATXβ, and ATXγ) and Novel Autotaxins (ATXδ and ATXε)

**DOI:** 10.1371/journal.pone.0130074

**Published:** 2015-06-17

**Authors:** Yasunori Tokuhara, Makoto Kurano, Satoshi Shimamoto, Koji Igarashi, Takahiro Nojiri, Tamaki Kobayashi, Akiko Masuda, Hitoshi Ikeda, Takeshi Nagamatsu, Tomoyuki Fujii, Junken Aoki, Yutaka Yatomi

**Affiliations:** 1 Department of Clinical Laboratory, The University of Tokyo Hospital, Tokyo, Japan; 2 CREST, Japan Science and Technology Corporation (JST), Saitama, Japan; 3 Department of Medical Technology, Kagawa Prefectural University of Health Sciences, Kagawa, Japan; 4 Department of Clinical Laboratory Medicine, Graduate School of Medicine, The University of Tokyo, Tokyo, Japan; 5 Department of Obstetrics and Gynecology, Graduate School of Medicine, The University of Tokyo, Tokyo, Japan; 6 Bioscience Division, Reagent Development Department, AIA Research Group, TOSOH Corporation, Kanagawa, Japan; 7 Laboratory of Molecular and Cellular Biochemistry, Graduate School of Pharmaceutical Sciences, Tohoku University, Miyagi, Japan; Showa University School of Pharmacy, JAPAN

## Abstract

**Background:**

Autotaxin (ATX) is a secreted enzyme that converts lysophosphatidylcholine to lysophosphatidic acid, a potent bioactive lipid mediator, through its lysophospholipase D activity. Although five alternative splicing isoforms of ATX have been identified as ATXα, ATXβ, ATXγ, ATXδ, and ATXε and the expression patterns of each isoform differ among several tissues, the clinical significance of each isoform remains to be elucidated.

**Methods:**

Anti-ATXβ and anti-ATXδ monoclonal antibodies were produced by immunization with recombinant human ATXβ and ATXδ expressed using a baculovirus system, respectively. We then developed enzyme immunoassays to measure the serum concentrations of “classical ATX” (ATXα, ATXβ, and ATXγ) and “novel ATX” (ATXδ and ATXε) antigens and evaluated the usefulness of these assays using human serum samples.

**Results:**

The with-run and between-run precision, interference, detection limit, and linearity studies for the present assay were well validated. In healthy subjects, the serum concentrations of classical ATX and novel ATX were significantly (*P* < 0.01) higher in women than in men, while the ratios of classical ATX or novel ATX to total ATX were not different between women and men. The concentrations of both classical ATX and novel ATX in normal pregnant subjects and patients with chronic liver diseases or follicular lymphoma were significantly higher than those in healthy subjects, while the ratio of both ATX isoforms to total ATX did not vary among these groups.

**Conclusions:**

We have developed a new enzyme immunoassay to determine the concentrations of classical ATX and novel ATX in human serum. These assays may be helpful for elucidating the distinct functional roles of each ATX isoform, which are largely unknown at present.

## Introduction

Autotaxin (ATX), which is known as ecto-nucleotide pyrophosphatase/phosphodiesterase family member 2, is one of the seven members of the pyrophosphatase/phosphodiesterase family. ATX is a secreted glycoprotein with a molecular weight of around 100 kDa that was originally isolated from the conditional medium of A2058 human melanoma cells [1]. ATX possesses lysophospholipase D (LysoPLD) activity and is known to act as an enzyme to produce lysophosphatidic acid (LPA), utilizing lysophosphatidylcholine (LPC) as a substrate [2–4]. Actually, we previously found that the ATX antigen concentration in human serum samples was strongly correlated with the serum LysoPLD activity and the plasma LPA concentrations [5].

LPA is a lipid mediator that exerts various biological actions, such as cellular proliferation, survival, migration, invasion, platelet activation and smooth muscle cell contraction [6–11], through six G-protein-coupled receptors (GPCRs), identified as LPA_1-6_. LPA acts on these GPCRs, which couple to several trimeric G proteins such as G_12/13_, G_i_, and G_q_, and subsequently stimulates the small GTPases Ras, Rho, and Rac and induces various pathophysiological actions, as described above [12–14].

Regarding the clinical significance of ATX, the concentration of serum ATX antigen was reportedly elevated in patients with chronic liver diseases (CLD) [5], follicular lymphoma (FL) [15], and pancreatic cancer [16] and was significantly higher in normal pregnant females than in non-pregnant healthy females [17].

To our knowledge, in all the clinical studies measuring ATX concentrations in human subjects published so far, the total ATX concentration has always been measured, even though ATX is known to have five alternative splicing isoforms [18,19]. The ATX gene is located on chromosome 8 in humans and has a highly complicated structure, containing 27 exons; thus, it could potentially have an even higher number of alternative splicing isoforms. Actually, three alternative splicing isoforms, i.e., ATXα, ATXβ, and ATXγ, were initially reported, and two novel alternative splicing ATX isoforms, i.e., ATXδ and ATXε, have also been recently identified. ATXδ and ATXε possess a 4-amino acid deletion in the L2 linker region of ATXβ and ATXα, respectively. Though all ATX isoforms have been shown to be enzymatically active, ATXβ and ATXδ are the major and stable isoforms and are present in a wide range of organisms, from fish to mammals [19].

**Fig 1:**
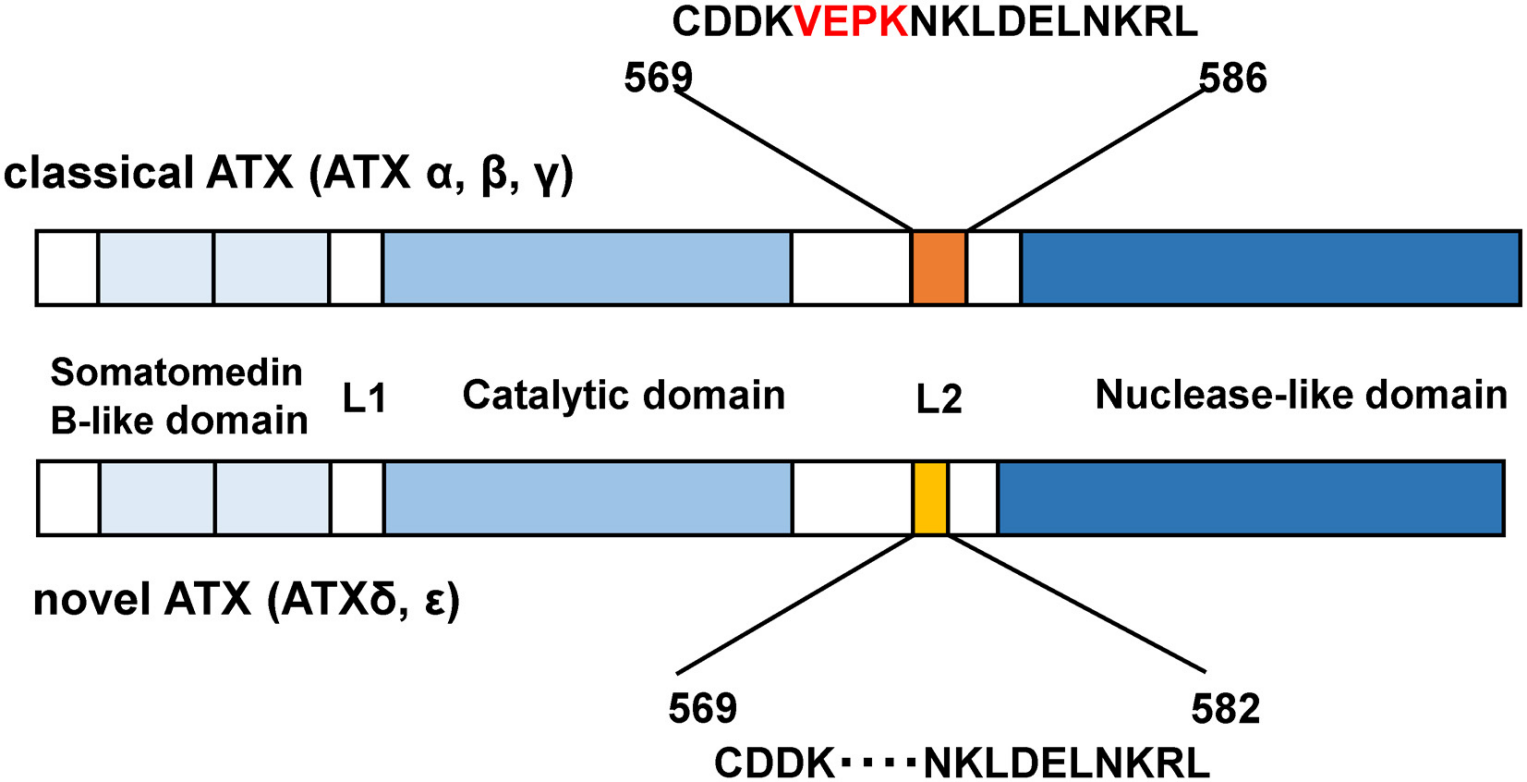
Schematic representation of “classical ATX” (ATXα, β, γ) and “novel ATX” (ATXδ, ε). The amino acid numbers corresponding to human ATXβ and ATXδ are shown, respectively. “Novel ATX” contains a 4-amino acid deletion in the L2 linker.

Considering the importance of ATX as an enzyme producing LPA, the measurement of serum total ATX concentrations is an important task. Furthermore, each ATX isoform might have some specific significance, and the concentrations of some ATX isoforms might be more useful for the diagnosis of various diseases than the total ATX concentration. Therefore, in this study, we developed enzyme immunoassays to measure the serum antigen concentration of ATXα, ATXβ, and ATXγ, which we defined as the “classical ATX” concentration, as well as that of ATXδ and ATXε, which we defined as the “novel ATX” concentration (Fig 1). We then used these immunoassays to measure these ATX concentrations in serum samples from healthy subjects, normal pregnant women, and patients with CLD, FL, or diabetes mellitus (DM).

## Materials and Methods

### Samples

The serum samples utilized in this study were the residuals of samples obtained for laboratory analyses. Written informed consent for sample analysis was obtained from each of the patients. As a control group, serum samples were collected from 57 healthy adult volunteers who had also provided written informed consent. To obtain the serum samples, whole blood specimens were directly put into glass tubes and left to stand for 15 min at room temperature to allow blood clots to form; the serum was then separated by centrifugation at 1500×g for 5 min. This study was approved by the Institutional Research Ethics Committee of the Faculty of Medicine, The University of Tokyo.

### Preparation of recombinant human ATX isoforms expressed in baculovirus system

Human cDNA for ATXβ was amplified using RT-PCR and human liver cDNA library as template DNA, based on sequence information in a database (GenBank [L46720]). Human ATXβ was introduced into the baculovirus transfer vector pFASTBac-HT, which includes a polyhistidine-tag at the NH2-terminus (Invitrogen, Carlsbad CA). ATXδ (GenBank [AAB00855]), which lacks amino acids 573–576 of human ATXβ (Fig 1), was constructed from a plasmid encoding ATXβ using inverted PCR and the KOD-Plus-Mutagenesis Kit (Toyobo, Tokyo, Japan) according to the manufacturer’s instructions, and then introduced to the baculovirus vector. The recombinant polyhistidine-tagged ATXβ (his ATXβ) and polyhistidine-tagged ATXδ (his ATXδ) were produced in Sf9 insect cells with the baculovirus system (Invitrogen, Carlsbad CA) and were purified using chelate column chromatography (BD Talon; BD Biosciences, San Jose, CA) and affinity chromatography with the NHS-activated HiTrap (GE Healthcare, Uppsala, Sweden) immobilized anti-ATX monoclonal antibody R10.7. Using this protocol, ATX could be eluted under mild acidic conditions (100 mmol/L citrate buffer, pH5.0) without the loss of LysoPLD activity.

### Production of anti-ATX and ATX isoform monoclonal antibodies

Anti-ATX monoclonal antibodies (R10.21 and R10.23), which recognized both ATXβ and ATXδ equivalently, were purified as described previously for the total ATX quantitative assay [5]. To generate antibodies specific to classical ATX and novel ATX, we prepared the peptide CDDKVEPKNKLDELNKRL (amino acids 569–586 of human ATX) and the 4 amino acid-deficient peptide CDDKNKLDELNKRL (Fig 1). These peptides were conjugated with maleimide-activated KLH (Pierce Biotechnology, IL), and the peptide-KLH conjugates were injected with Freund complete adjuvant into rats, yielding R4+4 mAb (anti-classical ATX antibody) and R4-127 mAb (anti-novel ATX antibody), respectively. The anti-ATX isoform mAbs were purified using a hybridoma culture supernatant and affinity chromatography with HiTrap Protein G (GE Health Science, WI).

### Combination screening of monoclonal antibodies for a 2-site immunoassay

Alkaline phosphatase (ALP)-labeled anti-ATX and ATX isoform mAbs were prepared for combination screening using a sandwich ELISA with a Peroxidase Labeling Kit-NH_2_ (Dojindo, Tokyo, Japan), according to the manufacturer’s protocol. Microtiter wells were coated with 100 ng of anti-ATX or ATX isoform mAbs and blocked with 3% BSA-TBS. After blocking, 50 ng of ATXβ or ATXδ was added to the wells, followed by addition of the ALP-labeled anti-ATX or ATX isoform mAbs. The antibody bound was detected by TMB peroxidase substrate (KPL, MD, USA). The detection of classical ATX was based on combination screening for anti-ATX and classical ATX mAbs using a 2-site immunoassay; we selected R10.23 for the solid-phase primary antibody and R4+4 for the enzyme-labeled secondary antibody. The detection of novel ATX was also based on combination screening for anti-ATX and novel ATX mAbs using a 2-site immunoassay; R4-127 was utilized for the solid-phase primary antibody, and R10.23 was utilized for the enzyme-labeled secondary antibody. The detection of total ATX was also based on combination screening for anti-ATX mAbs using a 2-site immunoassay; R10.23 was utilized for the solid-phase primary antibody and R10.21 was utilized for the enzyme-labeled secondary antibody [5].

### Western blotting

To validate the specificity of the newly developed antibodies, we performed a western blot analysis, as follows. The purified recombinant proteins ATXβ and ATXδ and serum samples were subjected to sodium dodecyl sulfate polyacrylamide gel electrophoresis (SDS-PAGE) and were transferred to a polyvinylidene fluoride membrane using a semi-dry blotter (Transblot SD Cell, Bio-Rad). The membranes were blocked for 2 hours at room temperature in Tris-buffered saline containing 3% powdered milk, then incubated with the anti-classical ATX antibody R4+4 (1 μg/mL) or the anti-novel ATX antibody R4-127 (1 μg/mL). After reaction with alkaline phosphatase-labeled anti-rat IgG, the monoclonal antibodies that were bound to the ATX isoforms were visualized using an enhanced chemiluminescence kit (CDP-STAR, Roche).

### Reactivity of mAbs to serum ATX isoforms

To evaluate the reactivity of mAbs to ATX in serum, R4+4 mAb, R4-127 and R10.23 immobilized on NHS-Hitrap column (GE Healthcare) were prepared. Normal human serum samples were applied to these columns and the pass-through fraction was collected. ATX concentration of this pass-through fraction was measured using total ATX assay, classical ATX assay and novel ATX assay described below. To evaluate the cross reactivity of ATX isoform mAbs to other species ATX in serum, mouse, rat, rabbit, dog and bovine serum were subjected to a western blot analysis with anti-classical ATX mAb (R4+4 mAb) and anti-novel ATX mAb (R4-127 mAb).

### Validation procedures

Three different pooled serum samples from healthy subjects and patients with CLD (with moderate and high ATX concentrations) were used to perform the within-run and between-run precision tests. For the within-run experiment, the measurement of 3 samples was replicated 10 times and the resulting coefficients of variation (CVs) were evaluated. For the between-run analysis, each sample was measured 4 times per run, and 5 runs were performed; the resulting CVs were then evaluated. To evaluate linearity, three different pooled serum samples (samples 1, 2, and 3) were diluted sequentially with the ATX-depleted serum (as described above) and were measured. The effects of various interfering materials contained within serum were also examined: the bilirubin F (free bilirubin), bilirubin C (conjugated bilirubin), and hemoglobin were checked using the Interference Check-A Plus kit (Sysmex, Kobe, Japan), and the chyle was checked using Intralipid (Terumo, Tokyo, Japan). The detection limit of each serum ATX assay was defined as the mean ± 3 SD for the blank. The zero calibrator measurement was replicated 10 times.

### Statistical analysis

The statistical significance of differences between two groups was determined using the Mann—Whitney *U* test, since the normality and equality of the variance were not validated in the various groups. All the data were expressed as the mean ± SD. A value of *P* < 0.05 was considered statistically significant. All the analyses were performed using StatFlex software (version 6.0; Artec Inc., Osaka, Japan).

## Results

### Validation of anti-classical and novel ATX antibodies employed

**Fig 2:**
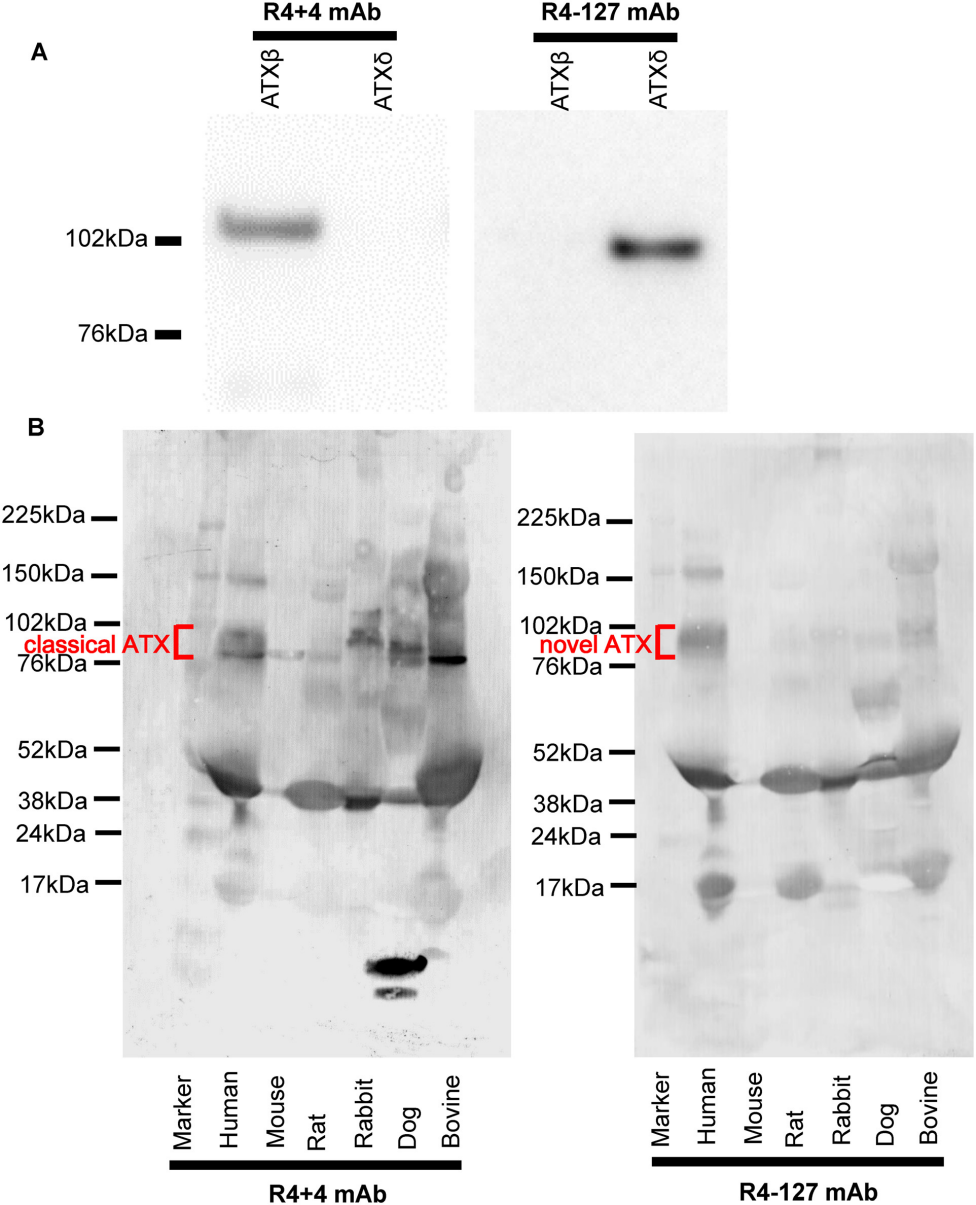
Specificity and cross reactivity of ATX isoform specific mAbs. (A) Purified hisATXβ and hisATXδ recombinant proteins (200 ng/lane) expressed using the baculovirus system were subjected to a western blot analysis with anti-classical ATX mAb (R4+4 mAb, left) and anti-novel ATX mAb (R4-127 mAb, right). (B) Human, mouse, rat, rabbit, dog and bovine serum (1 μL/lane) were subjected to a western blot analysis with anti-classical ATX mAb (R4+4 mAb, left) and anti-novel ATX mAb (R4-127 mAb, right).

To validate the specificity of R4+4 mAb and R4-127 mAb, purified recombinant ATXβ and ATXδ were subjected to SDS-PAGE and immunoblotting with R4+4 mAb or R4-127 mAb (Fig 2A). The R4+4 mAb was able to bind specifically to ATXβ, while the R4-127 mAb was able to bind specifically to ATXδ, indicating that the R4+4 and R4-127 mAbs established in this study could specifically recognize the targeted epitopes on classical ATX and novel ATX, respectively.

Human, mouse, rat, rabbit, dog and bovine serum were subjected to a western blot analysis to validate specificity of the ATX isoform mAbs in serum samples and to evaluate the cross reactivity of ATX isoform mAbs. As shown in Fig 2B, ATX isoform mAbs could detect ATX in human serum samples. Regarding the cross reactivity, only bovine ATX was detected by anti-classical ATX mAb (R4+4) and anti-novel ATX mAb (R4-127), respectively (Fig 2B). These results indicated that the classical and novel ATX assays are not useful for the evaluation for animal ATXs.

### Development of automated immunoassay for quantitative determination of ATX isoforms

An automated immunoassay for the quantification for each ATX isoform was established in the same manner as that used for the total ATX assay, and human serum samples were assayed using an automated immunoassay analyzer (AIA-system; TOSOH, Tokyo, Japan), as previously described [5]. Briefly, the antigen concentrations of classical ATX and novel ATX in the sera were determined using a specific 2-site enzyme immunoassay, and the combinations of the solid-phase primary antibody and the enzyme-labeled secondary antibody were described in MATERIALS AND METHODS. The AIA-system includes automated specimen dispensation, incubation of the reaction cup, a bound/free washing procedure, 4-methylumbelliferyl phosphate substrate dispensation, fluorometric detection, and a result report. After setting the serum samples in the AIA-system, 10 μL for classical ATX assay and 40 μL for novel ATX assay were applied to reaction cup with 140 μL dilution buffer for classical ATX assay and 110 μL for novel ATX assay. The antigen—antibody reaction time is 10 min, and the first result is reported within 20 min; the throughput of the system is 200 samples/h using the AIA-2000 system.

**Fig 3:**
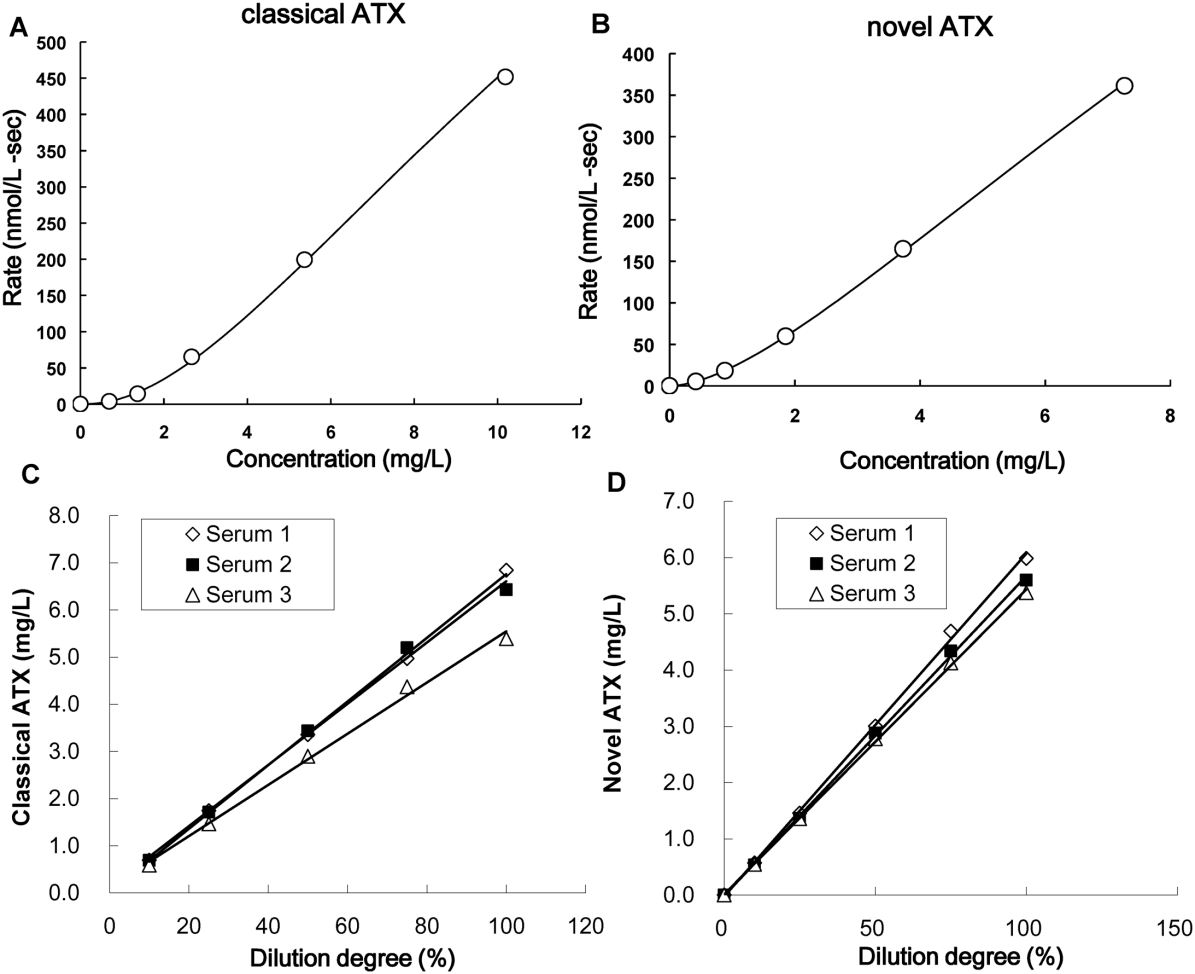
Development of the automated immunoassay for quantitative determination of ATX isoforms. (A, B) The calibration curves of the classical (A) and novel (B) ATX assays. Rate means the production per second of 4-Methylumbelliferone from 4-methylumbelliferyl phosphate by alkaline phosphatase. (C, D) Dilution linearity of the serum ATX isoform assays. Three different pooled serum samples (samples 1, 2, and 3) were diluted and measured using the classical (C) and novel (D) ATX assays.

Recombinant human ATXβ and ATXδ were produced as described above, and ATX-depleted serum was processed using an R10.23 (anti-ATX antibody, described below)-immobilized column, which was prepared using NHS-activated HiTrap. A 6-point calibrator set was prepared by spiking the purified recombinant ATXβ into the ATX-depleted serum at 0, 0.691, 1.372, 2.667, 5.373, and 10.19 mg/L. This calibrator set was used for the following classical ATX quantitative assay. In the same manner, a 6-point calibrator set for the novel ATX quantitative assay was prepared by spiking the purified recombinant ATXδ at 0, 0.420, 0.886, 1.857, 3.731, and 7.268 mg/L. The calibration curves were shown in Fig 3A and 3B.

The selectivity of the antibodies was also tested with the ELISA, using R4+4, R4-127 and R10.23 immobilized column (GE Healthcare). The concentration of classical ATX in the pass-through fractions of the R4+4 immobilized column was 0.016 ± 0.023 mg/L, while that of novel ATX in the pass-through fractions of the R4-127 immobilized column was 0.002 ± 0.001 mg/L (Table 1). Both the concentrations of classical and novel ATX in the pass-through fractions of R10.23 immobilized column were less than the minimum detection concentrations (Table 2). These results validated the specificity of each ATX isoform assay.

**Table 1:** The concentrations of total ATX, classical ATX, and novel ATX in sera adsorbed by an ATX isoform-specific mAb.

Immobilized mAb	Total ATX, mg/L	Classical ATX, mg/L	Novel ATX, mg/L
R4+4	0.222 ± 0.058	0.016 ± 0.023	0.222 ± 0.057
R4-127	0.542 ± 0.104	0.531 ± 0.105	0.002 ± 0.001

The mean ± SD (n = 28) are shown.

**Table 2:** The concentrations of total ATX, classical ATX, and novel ATX in sera before and after adsorption by R10.23.

Immobilized mAb	Total ATX, mg/L	Classical ATX, mg/L	Novel ATX, mg/L
Before adsorption	0.907 ± 0.332	0.532 ± 0.186	0.253 ± 0.099
After adsorption	undetectable	undetectable	undetectable

The mean ± SD (n = 20) are shown.

### Within-run and between-run precision of the serum ATX isoform assays

We first examined the within-run and between-run precision of our new serum ATX isoform assays. The mean ± SD values for the within-run study of the classical ATX assay were 0.641 ± 0.010, 2.369 ± 0.060, and 6.314 ± 0.180 mg/L, while those for the between-run study were 0.610 ± 0.019, 2.206 ± 0.079, and 6.179 ± 0.203 mg/L. The within-run and between-run CVs ranged from 1.94% to 2.86% and from 3.06% to 3.59%, respectively. The mean ± SD values for the within-run study of the novel ATX assay were 0.209 ± 0.010, 1.751 ± 0.030, and 5.784 ± 0.100 mg/L, while those for the between-run study were 0.208 ± 0.004, 1.750 ± 0.059, and 5.840 ± 0.099 mg/L. The within-run and between-run CVs ranged from 1.71% to 2.46% and from 1.69% to 3.19%, respectively.

### Interference studies for serum ATX isoform assays

Various serum ingredients that could interfere with the measurement were added to pooled serum samples (1:9 volume [bilirubin C, bilirubin F, and hemoglobin], and 1:5 volume [intralipid]) and the ATX isoform concentrations were measured. The addition of up to 199 mg/L of bilirubin C, 174 mg/L of bilirubin F, 4582 mg/L of hemoglobin, or 16667 mg/L of intralipid did not affect the measurements in either the classical or the novel ATX assay.

### The lower and upper detection limits of the serum ATX isoform assays

The lower detection limits of the classical and novel ATX assays were defined as the mean ± 3 SD for a blank. The zero calibrator measurement was replicated 10 times; the mean ± SD of the classical ATX assay was 0.009 ± 0.006 mg/L, and the minimum detection limit of this assay was estimated to be 0.027 mg/L. Meanwhile, the mean ± SD of the novel ATX assay was 0.004 ± 0.003 mg/L, and the minimum detection limit of this assay was estimated to be 0.013 mg/L. The upper limits of the classical and novel ATX assays were defined by the measurement of samples with high ATX concentrations, which was replicated 5 times. The mean ± SD of the classical ATX assay was 9.246 ± 0.094 mg/L and the percent recovery to theoretical concentration was 92.7% (1.01%CV). Meanwhile, the mean ± SD and the recovery of the novel ATX assay were 6.826 ± 0.093 mg/L and 99.2% (1.37%CV). The upper limits of the classical and novel ATX assay were considered to be 9.971 mg/L and 6.881 mg/L, respectively.

### Linearity of the serum ATX isoform assay

Three different pooled serum samples with high concentrations of ATXβ and ATXδ were diluted with ATX-depleted human serum and measured for ATXβ and ATXδ, respectively. The criteria for the recovery of each diluted sample concentration was 85%–115%. Well-fitted regression lines passing through all the measurement points for the classical and novel ATX assays were obtained for all the samples (Fig 3C and 3D). These results demonstrated that the ATX isoform assays met the acceptance criteria.

### ATX isoform concentrations in sera from healthy subjects

**Fig 4:**
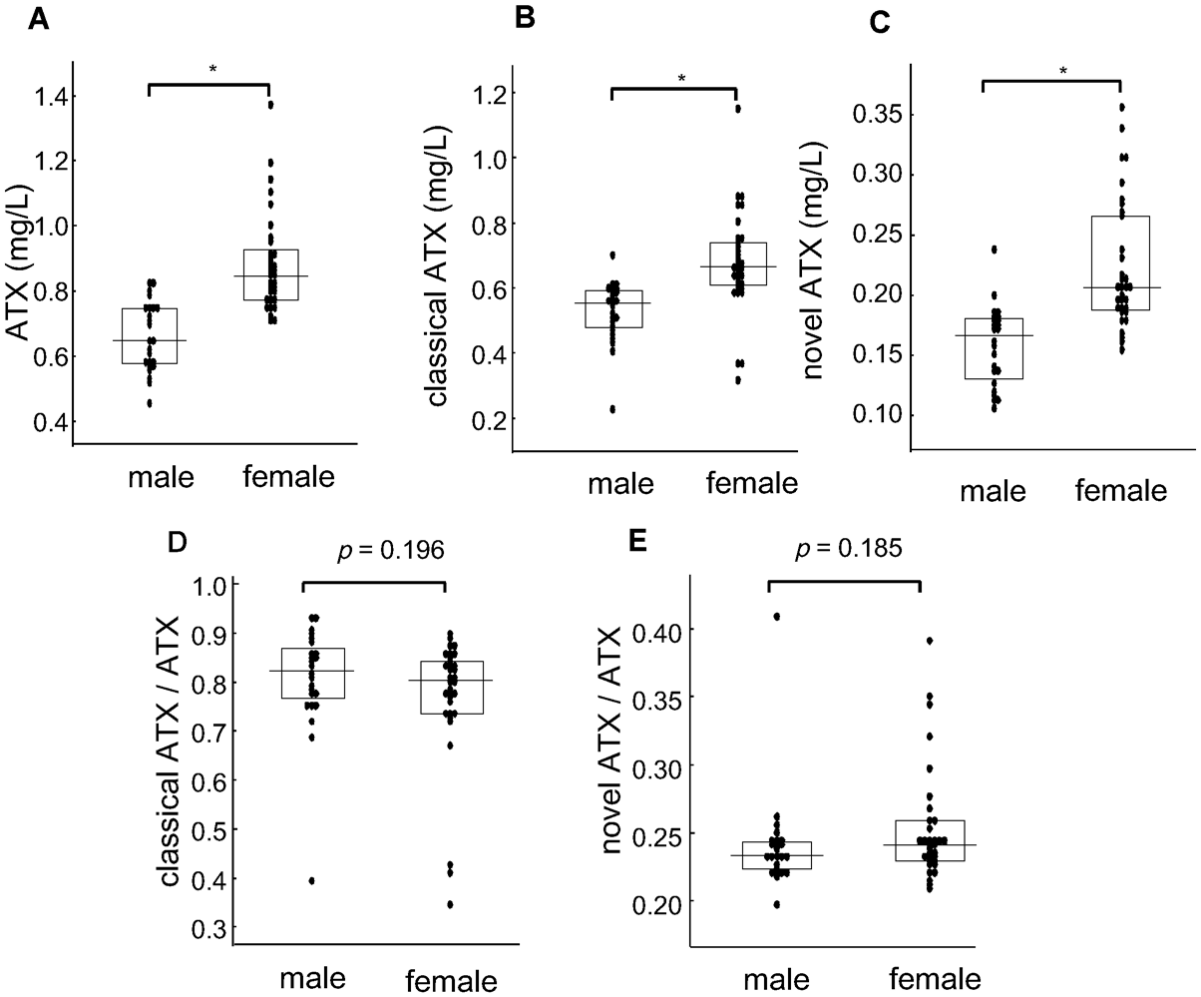
Concentration of ATX isoform in sera from healthy subjects. The concentration of total ATX (A), classical ATX (B), and novel ATX (C) antigen and the content ratio of classical ATX (D) and novel ATX (E) relative to total ATX was examined using the sera of healthy subjects (24 men and 33 women). *Statistically significant, as determined using the Mann-Whitney *U* test (*P* < 0.01).

The concentrations of total ATX and each ATX isoform antigen were measured for human sera obtained from 57 (24 men and 33 women) healthy subjects (Fig 4). The mean ± SD of the total ATX, classical ATX, and novel ATX antigen concentrations for the 57 healthy subjects were 0.788 ± 0.173 mg/L, 0.612 ± 0.151 mg/L, and 0.196 ± 0.056 mg/L, respectively, and the central 95^th^ percentile reference intervals for the total ATX, classical ATX, and novel ATX antigen concentrations were 0.510–1.205 mg/L, 0.312–0.905 mg/L, and 0.113–0.340 mg/L, respectively. The concentrations of total ATX, classical ATX, and novel ATX were significantly higher for female subjects than for male subjects (*P* < 0.01) (Fig 4A, 4B, and 4C), while the ratio of the classical ATX content to the total ATX content showed no significant difference between women and men (*P* = 0.196) (Fig 4D). Similar results were obtained for the ratio of novel ATX content to the total ATX content (*P* = 0.185) (Fig 4E).

### ATX isoform concentrations in sera from patients with chronic liver diseases, follicular lymphoma, and diabetes and cases with normal pregnancy

We previously reported that the concentration of serum total ATX antigen in patients with CLD and FL was significantly higher than that in healthy subjects [5,15], and the serum total ATX level was significantly higher in normal pregnant females than in non-pregnant healthy females [17]. Therefore, we next measured the concentrations of classical ATX and novel ATX antigens in sera from normal pregnant women and patients with CLD, FL, or DM. We then compared the serum ATX isoform antigen levels among the groups according to sex (Table 3). In whole subjects, the concentrations of the serum total, classical and novel ATX antigens were significantly higher in females than in males (Table 3). The concentrations of the serum ATX isoform antigens in normal pregnant females and patients with CLD or FL were significantly higher than those in the healthy subjects, while the ratio of each ATX isoform content to the total ATX content was similar among the groups (Table 3).

**Table 3:** The concentrations of the serum total ATX, classical ATX, and novel ATX antigens in males and females.

Diagnosis	total ATX, mg/L	classical ATX, mg/L	novel ATX, mg/L
		(classical ATX / ATX)	(novel ATX / ATX)
Whole subjects			
Male	0.979 ± 0.579	0.788 ± 0.486	0.244 ± 0.155
(n = 76)		(0.811 ± 0.119)	(0.246 ± 0.042)
Female	1.493± 0.980*	1.158 ± 0.815*	0.366 ± 0.236*
(n = 98)		(0.776 ± 0.120)	(0.248 ± 0.049)
Healthy subjects			
Male	0.658 ± 0.104	0.527 ± 0.094	0.159 ± 0.033
(n = 24)		(0.805 ± 0.110)	(0.241 ± 0.038)
Female	0.883 ± 0.150	0.673 ± 0.155	0.224 ± 0.054
(n = 33)		(0.766 ± 0.132)	(0.253 ± 0.042)
CLD			
Male	1.365 ± 0.767^†^	1.129 ± 0.645^†^	0.344 ± 0.201^†^
(n = 28)		(0.828 ± 0.072)	(0.249 ± 0.033)
Female	1.463 ± 0.667^‡^,	1.182 ± 0.582^‡^	0.410 ± 0.203^‡^
(n = 17)		(0.803 ± 0.114)	(0.275 ± 0.049)
FL			
Male	0.950 ± 0.273^†^	0.703 ± 0.198^†^	0.237 ± 0.092^†^
(n = 10)		(0.760 ± 0.145)	(0.246 ± 0.047)
Female	1.280 ± 0.472^‡^	0.973 ± 0.364^‡^	0.331 ± 0.110^‡^
(n = 15)		(0.762 ± 0.093)	(0.263 ± 0.055)
DM			
Male	0.778 ± 0.289	0.616 ± 0.206	0.196 ± 0.096
(n = 14)		(0.822 ± 0.180)	(0.247 ± 0.060)
Female	0.904 ± 0.274	0.772 ± 0.212	0.202 ± 0.084
(n = 8)		(0.862 ± 0.041)	(0.216 ± 0.056)
Normal pregnancy	2.637 ± 1.178^‡^	2.014 ± 1.086^‡^	0.599 ± 0.300^‡^
(n = 25)		(0.752 ± 0.132)	(0.224 ± 0.036)

CLD, chronic liver diseases; FL, follicular lymphoma; DM, diabetes mellitus.

* *p* < 0.01 vs Whole male subjects.

^†^ *p* < 0.01 vs Healthy male subjects.

^‡^ *p* < 0.01 vs Healthy female subjects.

## Discussion

In this study, we established enzyme immunoassays for the quantitative determination of classical ATX isoforms (ATXα, ATXβ, and ATXγ) and novel ATX isoforms (ATXδ and ATXε). We then used these immunoassays to measure the antigen levels in human serum samples using an automated analyzer. Based on the within-run and between-run precision, interference, detection limit, and linearity results obtained in the present study, we speculate that these assays for measuring classical ATX and novel ATX antigens can be available for clinical laboratory testing in the future. In healthy subjects, the serum antigen levels of classical ATX and novel ATX were significantly higher in women than in men (Fig 4B and 4C). Moreover, the serum ATX isoform antigen levels in normal pregnant women and patients with CLD or FL were significantly higher than those in the healthy subjects (Table 3). We also showed that the classical and novel ATX content ratios for ATX were similar when compared according to sex and the various disease groups.

Classical ATX and novel ATX are widely expressed in human tissues, with relatively high mRNA levels of classical ATX seen in the brain, placenta, ovary, and small intestine [20] and of novel ATX seen in the small intestine and spleen [19]. Hashimoto *et al*. reported that novel ATX accounted for up to 30% of the total ATX mRNA in various human tissues [19]. In the present study, the ratio of novel ATX antigen to total ATX was 0.241 ± 0.038 in healthy males and 0.253 ± 0.042 in healthy females (Fig 4D and 4E); these results were almost concordant with the expression levels of ATX in the examined tissues. The ratio of novel ATX to total ATX did not vary greatly between males and females or among subjects with various diseases (Table 3).

Although the biochemical characteristics of classical ATX and novel ATX are almost the same and we failed to find any significant differences between classical and novel ATX in this study, their expression patterns reportedly differ in human tissues and cell lines [19]. Since the expression of ATX is increased in some specific cancers, such as breast cancer, glioblastoma, teratocarcinoma [20], non-small-cell lung cancer [21], thyroid carcinoma [22], and melanoma [23], it is possible that the serum ATX isoform concentration might change in certain cancers originating from particular tissues or cells. Further studies are needed to elucidate the clinical usefulness of measuring ATX isoforms.

In summary, we developed a new enzyme immunoassay for the quantitative determination of classical ATX and novel ATX isoforms, in addition to a total ATX immunoassay. Although no remarkable differences in the ratio of each ATX isoform relative to the total ATX content were seen in patients with CLD, FL, or DM in the present study, these assays might help to elucidate the clinical significance of classical ATX and novel ATX isoforms in future studies.
